# Surface‐Based Connectivity Integration: An atlas‐free approach to jointly study functional and structural connectivity

**DOI:** 10.1002/hbm.25447

**Published:** 2021-05-06

**Authors:** Martin Cole, Kyle Murray, Etienne St‐Onge, Benjamin Risk, Jianhui Zhong, Giovanni Schifitto, Maxime Descoteaux, Zhengwu Zhang

**Affiliations:** ^1^ Department of Biostatistics and Computational Biology University of Rochester Rochester New York USA; ^2^ Department of Physics and Astronomy University of Rochester Rochester New York USA; ^3^ Sherbrooke Connectivity Imaging Laboratory (SCIL) Université de Sherbrooke Québec Canada; ^4^ Department of Biostatistics and Bioinformatics Emory University Atlanta Georgia USA; ^5^ Department of Imaging Sciences University of Rochester Rochester New York USA; ^6^ Department of Neurology University of Rochester Rochester New York USA; ^7^ Department of Statistics and Operations Research University of North Carolina at Chapel Hill North Carolina USA

**Keywords:** connectome integration, continuous connectome, diffusion MRI, functional MRI, white surface

## Abstract

There has been increasing interest in jointly studying structural connectivity (SC) and functional connectivity (FC) derived from diffusion and functional MRI. Previous connectome integration studies almost exclusively required predefined atlases. However, there are many potential atlases to choose from and this choice heavily affects all subsequent analyses. To avoid such an arbitrary choice, we propose a novel atlas‐free approach, named Surface‐Based Connectivity Integration (SBCI), to more accurately study the relationships between SC and FC throughout the intra‐cortical gray matter. SBCI represents both SC and FC in a continuous manner on the white surface, avoiding the need for prespecified atlases. The continuous SC is represented as a probability density function and is smoothed for better facilitation of its integration with FC. To infer the relationship between SC and FC, three novel sets of SC‐FC coupling (SFC) measures are derived. Using data from the Human Connectome Project, we introduce the high‐quality SFC measures produced by SBCI and demonstrate the use of these measures to study sex differences in a cohort of young adults. Compared with atlas‐based methods, this atlas‐free framework produces more reproducible SFC features and shows greater predictive power in distinguishing biological sex. This opens promising new directions for all connectomics studies.

## Graphical abstract

There has been increasing interest in jointly studying structural connectivity (SC) and functional connectivity (FC) derived from diffusion and functional MRI. In the following article, we propose a novel atlas‐free approach, named Surface‐Based Connectivity Integration (SBCI), to more accurately study the relationships between SC and FC throughout the intra‐cortical gray matter. Compared with atlas‐based methods, the atlas‐free framework produces more reproducible SC‐FC coupling features and shows greater predictive power in distinguishing biological sex. This opens promising new directions for all connectomics studies.

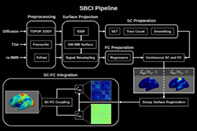

## 1 INTRODUCTION

Brain connectivity is an intriguing and quickly expanding research field (Eyewire & Blake, 1997; Glasser, Smith, et al., 2016; Park & Friston, 2013; Shi & Toga, 2017; Smith et al., 2015). With recent advances in magnetic resonance imaging (MRI) techniques, we are able to noninvasively probe the human brain at higher resolutions than ever before (Glasser, Coalson, et al., 2016) and construct different types of connectomes. Among them, two imaging modalities, diffusion MRI (dMRI) and functional MRI (fMRI), and their corresponding brain connectivities are particularly prominent. While dMRI measures the restriction of isotropic diffusive water movement (Basser, Mattiello, & LeBihan, 1994) and can be used to infer structural connectivity (SC) (Sporns, 2013a), fMRI measures the blood oxygen level dependent (BOLD) signal, deemed as a proxy for neurovascular coupling due to large‐scale neural activation (Ogawa, Lee, Kay, & Tank, 1990) and can be used to infer functional connectivity (FC) (Sporns, 2013b).

Both SC and FC play important roles in understanding the human brain (Sporns, 2013b; Zimmermann, Griffiths, & McIntosh, 2018). As a result of the anatomy of white matter (WM), dMRI can be used to estimate the restricted diffusive patterns of water throughout the brain by applying magnetic gradients of multiple strengths in many directions (Li, Shi, & Toga, 2016). Diffusion signals are then reconstructed per voxel and represented as fiber orientation distribution functions (fODFs), which can then be fitted to tractography algorithms to estimate the locations of WM connections (Basser et al., 1994; Bastiani et al., 2012; Descoteaux et al., 2009; Girard, Whittingstall, Deriche, & Descoteaux, 2014; Tournier, Calamante, & Connelly, 2012). SC aims to measure the extent to which brain regions are connected by WM fiber bundles, where traditionally regions are obtained based on a parcellated atlas (Zhengwu Zhang, Allen, Zhu, & Dunson, 2019; Zhengwu Zhang et al., 2018). In the context of image analysis, we are limited to inferring SC from the streamlines connecting any pair of brain regions constructed by tractography algorithms (Girard et al., 2014; Maier‐Hein et al., 2017; Thomas et al., 2014) due to MRI acquisition limitations, such as low spatial resolution and signal‐to‐noise ratio. On the other hand, FC is obtained from calculating the correlation of BOLD signals between different brain regions. Differences in magnetic susceptibilities between oxygenated and deoxygenated blood give rise to the BOLD signal after neural stimulation has occurred in a region (Ogawa et al., 1990). BOLD imaging produces a time series of these susceptibility changes and is thought to represent the overall blood oxygenation exchange in each voxel as a function of time.

Although most existing connectivity studies explore SC or FC independently, there is increasing interest in exploring SC and FC together, referred to as SC and FC integration in this paper. Much of this previous integration workfalls roughly into three broad classes: *prediction*, *modeling*, and *fusion*. As one of the earliest papers studying the relationships between SC and FC, Honey et al. (2009) demonstrated that brain regions that are directly structurally connected have higher FC than those regions that are not directly connected, leading to a series of studies that attempted to *predict* an individual's FC directly from SC (Chamberland et al., 2017; Deligianni et al., 2013; Goñi et al., 2014; Higgins, Kundu, & Guo, 2018; Honey, Thivierge, & Sporns, 2010; Li, Shafipour, Shafipour, & Zhang, 2019; Messé, Rudrauf, Giron, & Marrelec, 2015). *Modeling* FC with neural spiking models by incorporating known SC as prior information has also drawn some attention recently. By assuming that SC and FC are highly correlated, it may be possible to derive FC patterns directly from neural spiking equations and SC to simulate hemodynamic response functions (HRFs) without collecting any functional data (Bassett, Zurn, & Gold, 2018; Iyer et al., 2013; Nakagawa, Jirsa, Spiegler, McIntosh, & Deco, 2013; P. Wang et al., 2019). Finally, connectivity *fusion* uses measurements from both dMRI and fMRI to derive new information about the brain (Bassett et al., 2018; Zhu et al., 2014). For example, Fan et al. (2016) built a novel brain atlas, called the Brainnetome atlas, using information from SC and FC, incorporating both structural and functional information across the brain.

These previous studies of SC and FC integration used different image resolutions, tractography algorithms, and brain parcellations to reconstruct the connectomes. Some found that the strength of FC is related to the anatomical pathways (SC) (F. D. Bowman, Zhang, Derado, & Chen, 2012; Goñi et al., 2014; Hermundstad et al., 2013; Van Den Heuvel, Mandl, Kahn, & Hulshoff Pol, 2009), while others concluded that the correlation between SC and FC is poor (Buckner, Krienen, & Yeo, 2013; Chamberland et al., 2017; Ghumman, Fortin, Noel‐Lamy, Cunnane, & Whittingstall, 2016). These heterogeneous findings lead to several fundamental questions we must consider when studying SC and FC jointly:

1. Which structure in the brain (e.g., gray matter, white matter, the white or pial surface) is the best place for exploring the relationships between SC and FC?
2. Which parcellation is most suitable for SC and FC integration?
3. How should intra‐cortical coupling between SC and FC be defined and how does such coupling vary across different populations?

Most existing studies use gray matter (GM) regions of interest (ROIs) as network nodes in deriving SC and FC. However, due to the limitations in dMRI acquisition (Reveley et al., 2015) and streamline reconstruction (Girard et al., 2014; Maier‐Hein et al., 2017; Thomas et al., 2014), using GM ROIs to build SC can result in biased SC estimation. For example, streamlines can stop prematurely in the WM or near the GM‐WM interface, superficial WM tracts can impede the construction of longer streamlines (Reveley et al., 2015), and the precision of streamline reconstruction is reduced due to partial volume effects (PVEs) in lower spatial resolution acquisitions (Tournier, Mori, & Leemans, 2011). Similarly, using GM ROIs as nodes in FC often requires volumetric smoothing and PVE correction, which reduces the spatial localization of BOLD signals and thus provides inaccurate FC estimation (Coalson, Van Essen, & Glasser, 2018).

Traditional construction of SC and FC often requires predefined parcellations/atlases for two primary reasons: (a) dimensionality reduction and (b) more straightforward interpretations of localized physical processes (Glasser, Coalson, et al., 2016). However, choosing an optimal brain parcellation for SC and FC integration is challenging due to the complicated nature of the brain. Parcellations derived from one modality (Gordon et al., 2014; Thomas Yeo et al., 2011) may not be suitable for studying others. As such, we currently do not know the most suitable parcellation to study SC and FC jointly.

Finally, the correlation or coupling strength between SC and FC has the potential to unlock some key insights on how brain structure collaborates with function. For example, it is natural to expect that such a correlation can be spatially different across brain regions and populations (Nakagawa et al., 2013). However, few studies have evaluated the relationship between SC and FC at high resolution, nor how this relationship may differ between different groups of subjects (e.g. males vs. females).

**FIGURE 1:**
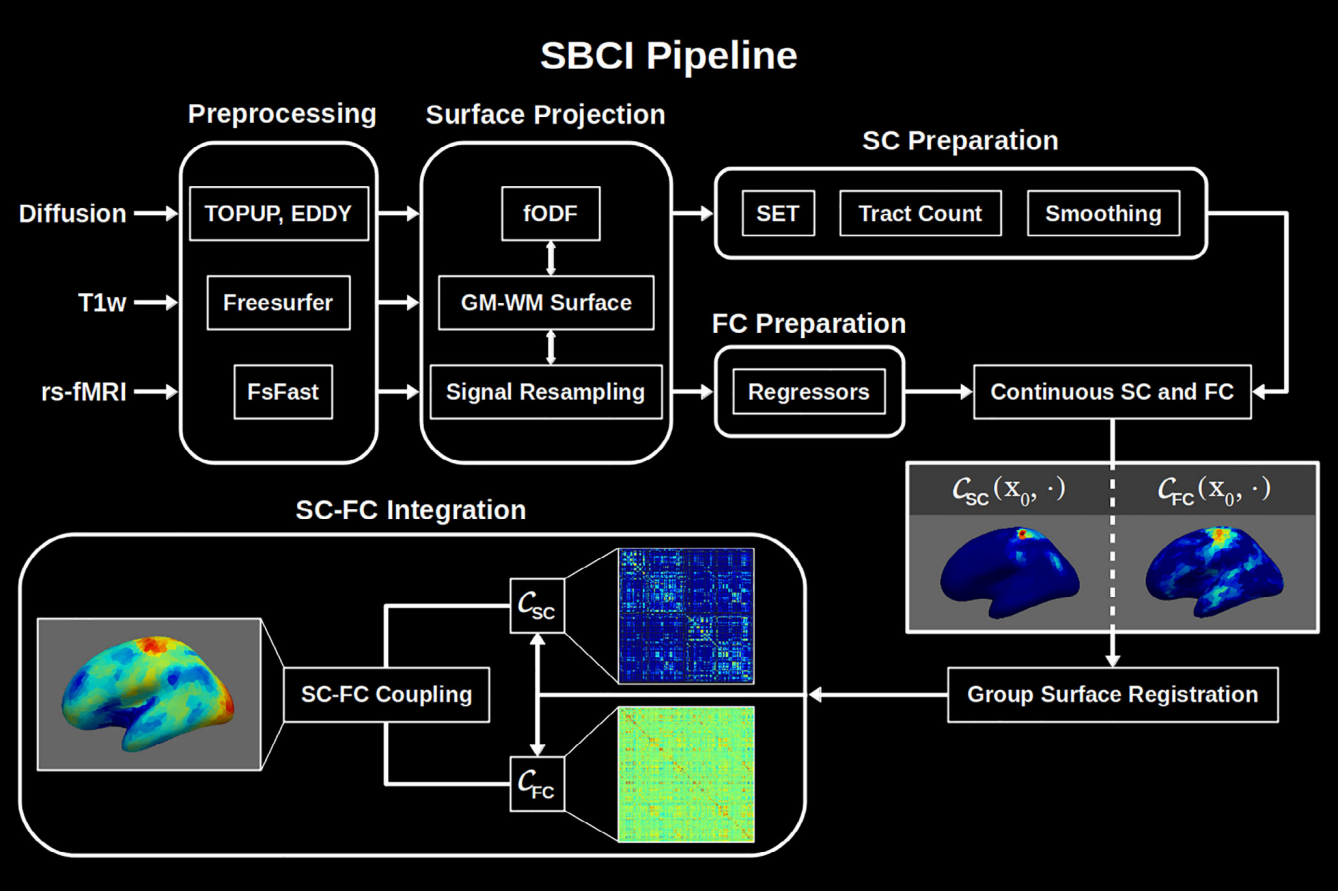
Flowchart of the SBCI pipeline. $C_{\mathrm{FC}}$, continuous FC; $C_{\mathrm{SC}}$, continuous SC; FC, functional connectivity; fODF, fiber orientation distribution function; FsFast, Freesurfer's Functional Analysis Stream; GM, gray matter; SBCI, surface‐based connectivity integration; SC, structural connectivity; SET, surface‐enhanced tractography; WM, white matter

With a comprehensive re‐thinking of these problems, we propose a novel atlas‐free approach, named Surface‐Based Connectivity Integration (SBCI), to facilitate the integrative analysis of SC and FC. In SBCI, we propose to use the white surface (the interface of white matter and gray matter) to build both SC and FC and represent them in a continuous fashion without the need of any pre‐specified brain parcellation. SBCI also gives three novel SC‐FC coupling strength measures to study the relationship between SC and FC across the brain's cortical surface. Figure 1 shows a systematic overview of the SBCI framework. Compared with existing work, SBCI has the following unique features:

- ***SBCImapsbothSCandFCtothewhitesurface***. The white surface is the interface between cortical GM and WM. Since diffusion signals are mostly in the WM regions and functional signals are more present in the GM regions, we posit that the white surface is the best place to study the integration of SC and FC. While many FC studies are based on the cortical surface (Coalson et al., 2018; Glasser, Coalson, et al., 2016), studies of the SC on this surface are still limited. Extending tractography reliably to the white surface comes with many challenges (Reveley et al., 2015). A novel algorithm called Surface‐Enhanced Tractography (SET) (St‐Onge, Daducci, Girard, & Descoteaux, 2018) overcomes many of these challenges by incorporating prior knowledge from the geometry of the white surface. Instead of using the unreliable dMRI signal near the white surface, SET uses a surface flow technique to model the superficial WM structure. In SET, all reconstructed streamlines intersect with the white surface.
- ***SBCItreatsSCandFCascontinuousfunctions***, ***avoidingtheneedforpre**‐**specifiedatlases***. For any two points on the white surface, SBCI defines connectivity strengths for SC and FC. We smooth the sparse SC to result in a continuous measure that facilitates integration with FC. Continuous treatment of SC and FC has two clear advantages in the study of SC‐FC integration: (a) connectomes can be represented at very high spatial resolution compared to current atlas‐based methods and (b) there is no need to rely on predefined parcellations to construct connectivity matrices, allowing for a more flexible and robust treatment of connectivity analysis (Messé, 2019).
- ***SBCIproducescontinuousanddiscreteSC‐FCcouplingfeaturesonthewhitesurface***. We define SC‐FC coupling (SFC) as the similarity between continuous SC and FC at any point on the white surface. SBCI gives three novel measures of different SFC features: continuous global coupling between SC and FC without a predefined parcellation, continuous local coupling within ROIs defined by a given parcellation, and discrete coupling with a given parcellation. Details are presented in Section 2.5.

In the following sections, we describe the details of the SBCI pipeline, experiments used to validate it, and an application of the SFC features as imaging markers to distinguish biological sex.

## 2 METHODS

### 2.1 Datasets

The HCP is the current gold standard for human brain connectivity mapping. In order to develop and validate the SBCI pipeline, we used the HCP Young Adult (HCPYA) and HCP Test–Retest (HCPTR) data. Data were downloaded from the ConnectomeDB website.

The data used in this paper include preprocessed T1‐weighted (T1w) and dMRI images and unprocessed resting‐state fMRI images from the HCPYA and HCPTR datasets. Full imaging acquisition information and minimal fMRI and dMRI image preprocessing steps are documented in Glasser et al. (2013). Briefly, all imaging was conducted on the 3T Siemens Connectom scanner (Erlangen, Germany). High‐resolution T1w anatomical images were acquired with the 3D MPRAGE (magnetization prepared rapid gradient echo) sequence with a slice acceleration factor of 2 using 0.7 mm isotropic resolution. Diffusion imaging was performed using a 2D spin‐echo EPI (echo planar imaging) sequence with approximately 90 diffusion directions at three nonzero b‐values (1,000, 2,000, and 3,000 s/mm^2^ ) each and 6 b0 reference scans at 1.25 mm isotropic resolution. A full diffusion MRI run includes 6 runs of about 9 min 50 s each, representing 3 gradient tables, with each table acquired once with right‐to‐left (RL) and left‐to‐right (LR) phase encoding polarities, respectively. Resting‐state functional imaging was performed using a 2D gradient‐echo EPI sequence with repetition time 720 ms, echo spacing 33.1 ms, and 2 mm isotropic resolution. Parallel imaging was enabled using a multiband acceleration factor of 8. Resting‐state fMRI scans were acquired in 4 runs of approximately 15 min each, with eyes open in a dark room. Runs alternated encoding polarities, resulting in two RL and two LR scans.

Our HCPTR data include 38 subjects with complete MR imaging data collected at Washington University in St. Louis as a follow‐up to an initial HCPYA scan, resulting in two full sets of imaging data (test and retest) for each subject. Our HCPYA data include 89 random subjects (46 females) from the S500 data release in the 26–30 year old group. We also tested SBCI in a few subjects collected in a Siemens MAGNETOM PrismaFit (Erlangen, Germany) scanner at the University of Rochester to verify that SBCI can give valid results in relatively low‐resolution MRI data (details are presented in Section S1; Data S1) and K. D. Murray et al. (2020)).

### 2.2 Image preparation and preprocessing

Diffusion image preprocessing steps including brain extraction, susceptibility‐induced distortion correction, motion correction, and eddy‐current distortion correction are performed using tools in FMRIB's software language (FSL) (Smith et al., 2004; Sotiropoulos et al., 2013; Woolrich et al., 2009). For both HCP datasets, we downloaded the diffusion data after eddy‐current correction. We begin with the T1w anatomical images after gradient correction and the raw rs‐fMRI. Additional diffusion processing are performed using a combination of tools from Mrtrix3 (https://www.mrtrix.org/), advanced normalization tools (ANTs) (Avants et al., 2011), and the Sherbrooke Connectivity Imaging Lab toolbox in Python (Scilpy) and included resampling to 1 mm isotropic resolution and building the fODFs to prepare for tractography.

The anatomical T1w processing includes registration to the high resolution diffusion images via ANTs and surface reconstruction using the recon_all tool available in Freesurfer (http://freesurfer.net/).

Resting‐state fMRI images are processed using Freesurfer's functional analysis stream (FsFast) which includes motion correction, brain masking, sampling to the surface (left and right), and surface smoothing with a Gaussian kernel with full width at half‐maximum (FWHM) *σ* in Freesurfer. Common nuisance variables are calculated in Freesurfer and include the WM signal, CSF signal, six motion parameters (three translational and three rotational), and the global signal (Murphy & Fox, 2017), where we use the top 5 principal components (PCs) for WM and CSF signals. More details about this regression can be found in Fox, Iaria, and Barton (2009); Murphy, Birn, and Bandettini (2013); and Murphy and Fox (2017). Additional processing details are available in Section S2 (Data S1).

### 2.3 Continuous functional connectivity on the white surface

During the FsFast pipeline, we map the volumetric BOLD signals to the subject's 32k white surface, resulting in a BOLD time series at each vertex on the surface meshes. The FC between any pair of vertices is usually calculated using a partial correlation between the two BOLD time series (Smith, 2012), controlling for confounding signals. Let $s_{v_{i}}\left(t\right)$ be the residual BOLD time series after regressing out nuisance variables at the *i*th vertex *v*_*i*_, and the FC between any vertex pair (*v*_*i*_, *v*_*j*_) is calculated as:

(1) $$FC_{\mathrm{ij}}=\left\{\begin{array}{ll} \text{corr}\left(s_{v_{i}},s_{v_{j}}\right), & \text{if}\;i\neq j\\ 0, & \text{if}\;v_{i}=v_{j} \end{array}\right.,$$

where we do not consider self‐interactions.

Although the FC obtained in (1) can be high resolution with dense surface meshes, it is still considered to be discrete. In our SBCI pipeline, we represent FC in a continuous fashion. Hence, we introduce the continuous FC concept with some mathematical formalism. We use the convention that a vertex is a location on the mesh grid and a point is any location on the surface. Let *Ω* be the union of two disjoint white surfaces of the brain; the continuous FC is represented as a symmetric function on the space *Ω* × *Ω*. For any pair of points (*x*,*y*) ∈ *Ω* × *Ω*, we define the continuous FC as $C_{\mathrm{FC}}\left(x,y\right)=\text{corr}\left(s_{x},s_{y}\right)$, where *s*_*x*_ and *s*_*y*_ are the BOLD time series at points *x* and *y*. We define the set of all possible FCs as $F_{\mathrm{FC}}=\left\{C_{\mathrm{FC}}:\Omega \times \Omega \mapsto \left[-1,1\right]:C_{\mathrm{FC}}\left(x,y\right)=0\;\text{if}\;x=y;\text{and}\;C_{\mathrm{FC}}\left(x,y\right)=C_{\mathrm{FC}}\left(y,x\right)\right\}$.

Now we have two major problems before getting the continuous FC. First, we only observe BOLD signals at discrete vertices (after the FsFast preprocessing), not every point on *Ω*. Second, the white surfaces between subjects are different, making any group‐wise analyses (analyses across multiple subjects) difficult. To solve these problems we inflate each white surface into a 2‐sphere, and with some abuse of notation also denote *Ω* as the union of two 2‐spheres $S_{1}^{2}\cup S_{2}^{2}$. The geometry of a 2‐sphere will more easily facilitate signal processing and inter‐subject alignment (Dale, Fischl, & Sereno, 1999; Fischl, Sereno, & Dale, 1999).

To obtain a BOLD signal for any point *x* ∈ *Ω*, we apply the following signal interpolation method on the 2‐sphere. Without loss of generality, assume $x\in S_{1}^{2}$ (the first 2‐sphere in *Ω*) and let *B*(*x*; *σ*) represent a neighborhood near *x* such that all vertices in *B*(*x*; *σ*) have geodesic distances to *x* less than *σ*. We have the BOLD time course *s*_*x*_ at *x* calculated as $s_{x}=\sum_{v'\in B\left(x;\sigma \right)}w_{\sigma }\left(x,v';\right)s_{v'}$, where *s*_*v*′_ are observed BOLD signals and *w*_*σ*_(*x*,*v*′) are the corresponding weights. *w*_*σ*_(*x*,*v*′) can be obtained from a truncated Gaussian or bi‐weight (quartic) kernel with bandwidth *σ* defined with a geodesic distance on the 2‐sphere, for example, $w_{\sigma }\left(x,v\prime \right)=\left(15/16\sigma \right){\left\{1-{\left(d\left(x,v\prime \right)/\sigma \right)}^{2}\right\}}^{2}I_{d\left(x,v\prime \right)<\sigma }$ (Risk & Zhu, 2019).

When multiple subjects are involved in the analysis, registration between subjects is necessary, i.e., finding correspondence among *Ω*_1_, …, *Ω*_*n*_ for *n* subjects. One of the advantages of inflating the original complex white surfaces to two 2‐spheres is that registration of signals on a 2‐sphere is much easier than on an irregular manifold space (Coalson et al., 2018; Glasser, Coalson, et al., 2016; Kurtek et al., 2010; Robinson et al., 2014). In this work, we used the alignment algorithm and registration results from Freesurfer. Other spherical registration algorithms have been proposed in the literature (Robinson et al., 2014).

### 2.4 Continuous structural connectivity on the white surface

In SBCI, we rely on two recent developments to reliably extend SC to the white surface and represent it in a continuous fashion. First, a recently published tractography algorithm called SET (St‐Onge et al., 2018) builds streamlines that extend through the white surface. Second, similar to the work of Moyer, Gutman, Faskowitz, Jahanshad, and Thompson (2017), we define the continuous SC by estimating a probability density function on *Ω* × *Ω*.

**FIGURE 2:**
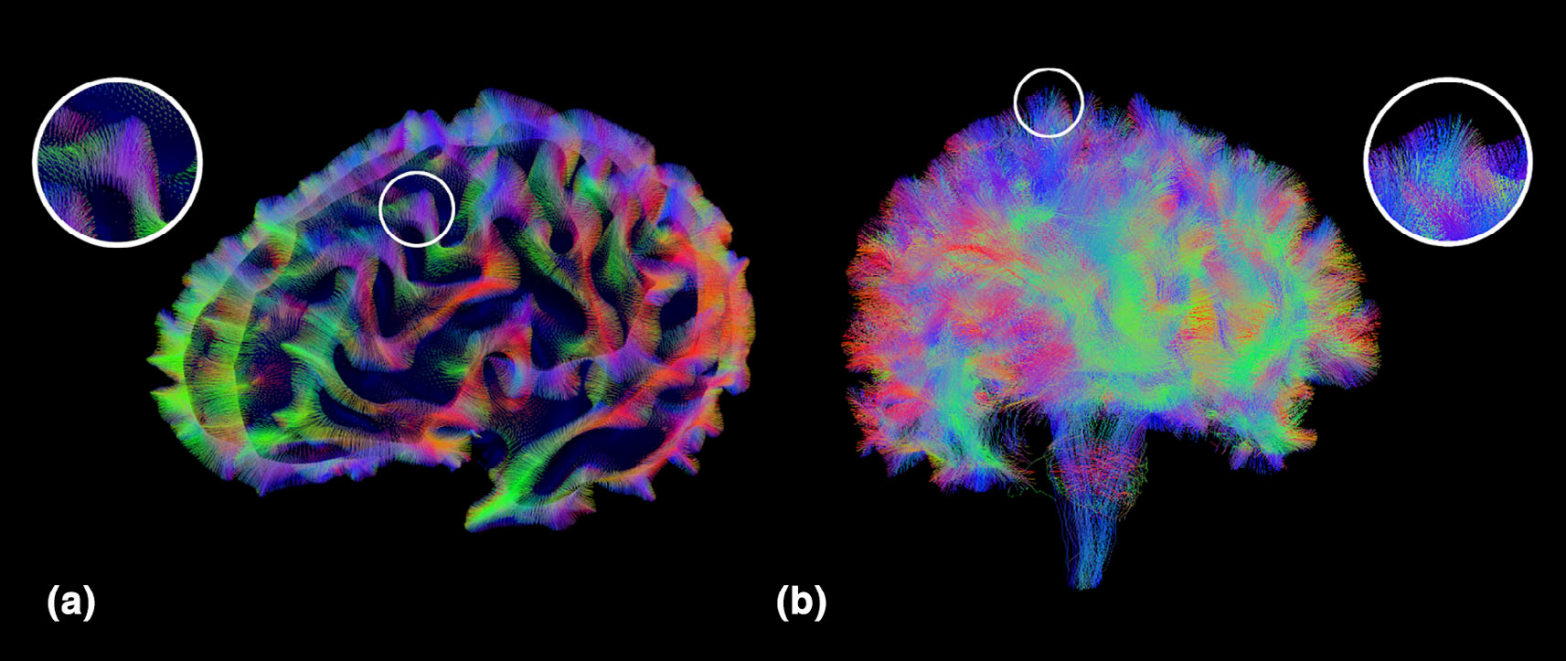
Tractography results from SET. (a) shows the surface flow to the white surface, and (b) shows the final tractography with the reconstructed fanning structure near the white surface

In SET, the white surface is used to initiate the flow with a parameter *t* controlling for the amount of flow into the WM, resulting in a surface beneath the white surface. Starting from the surface at *t* > 0, we initialize streamlines using *N*_*s*_ seed points on the surfaces and propagate them using the particle filtering technique (PFT) (Girard et al., 2014). Figure 2 illustrates some results from the SET pipeline, where (a) shows the initial white matter segments constructed by SET and (b) shows the final tractography result. Experiments have shown that SET decreases SC gyral biases and better approximates the underlying anatomy by using a more stringent assumption in regions where dMRI signals are less informative (St‐Onge et al., 2018).

We deviate from the traditional parcellation‐based approach to SC and define a continuous SC similar to the continuous FC described in Section 2.3. Maintaining both SC and FC in a comparable continuous framework allows for a more robust treatment of the integration of the two modalities. For any two distinct points *x* and *y* on the brain surfaces, we define SC as $C_{\mathrm{SC}}\left(x,y\right)\in {\mathbb{R}}_{+}\cup \left\{0\right\}$, where SC is the probability density function representing the likelihood that *x* and *y* are structurally connected by WM fibers. $C_{\mathrm{SC}}$ is a symmetric function defined on *Ω* × *Ω* and the set of all SC functions can be denoted as $F_{\mathrm{SC}}=\{C_{\mathrm{SC}}:\Omega \times \Omega \mapsto {\mathbb{R}}_{+}\cup \left\{0\right\}:C_{\mathrm{SC}}\left(x,y\right)=0\;\text{if}\;x=y$;$\;C_{\mathrm{SC}}\left(x,y\right)=C_{\mathrm{SC}}\left(y,x\right)\;\text{and}\int \int_{\Omega \times \Omega }C_{\mathrm{SC}}\left(x,y\right)\text{dxdy}=1\}$.

The limited number of streamlines (typically a few million) constructed with SET gives us a discrete and sparse sampling of $C_{\mathrm{SC}}$ (refer to Figure 4 panel (a)). In contrast, FC has non‐zero values for nearly every connection. In order to more fairly compare and integrate structural and functional connectivities, we estimate a smooth and dense SC using kernel density estimation (KDE) on *Ω* × *Ω*. Assume that we have a symmetric kernel *k*_*h*_ defined as a mapping from *Ω* × *Ω* to ℝ_+_ ∪ {0} with a bandwidth parameter *h*. The smoothed SC under a standard KDE procedure is given by:

(2) $${\hat{C}}_{\mathrm{SC}}\left(x,y\right)=N^{-1}\sum_{i=1}^{N}k_{h}\left(\left(x,y\right);\left(x_{i},y_{i}\right)\right),$$

where *N* is the total number of observed streamlines, and (*x*_*i*_, *y*_*i*_) represents the endpoints of the *i*th observed streamline. Now, we must define an appropriate kernel function *k*_*h*_ on *Ω* × *Ω*. Similar to Moyer et al. (2017) and Risk and Zhu (2019), we begin by defining a symmetric heat kernel (Hartman & Watson, 1974) on a 2‐sphere as:

(3) $$f_{h}\left(x;\mu \right)=A_{2}^{-1}\sum_{m=0}^{\infty }N_{m}\exp\left[-m\left(m+1\right)h\right]P_{m}\left(\left\langle x,\mu \right\rangle \right),$$

where $\mu \in S^{2}$ and *h* ∈ ℝ_+_ represent the mean and bandwidth, respectively, *A*_2_ = 4*π* (the area of $S^{2}$), *m*(*m* + 1) are the eigenvalues of the Laplacian on $S^{2}$ for *m* = 0, 1, …, ∞, *P*_*m*_ is the Legendre polynomial of order *m* for ℝ^3^, *N*_*m*_ equals 2*m* + 1, the number of linearly independent homogeneous spherical harmonics of degree *m* in *ℝ* ^3^, and ⟨,⟩ indicates the inner product of two elements on $S^{2}$. Since $\Omega =S_{1}^{2}\cup S_{2}^{2}$, we extend *f*_*h*_ to *Ω* trivially by letting *f*_*h*_(*x*; *μ*) = 0 if *x* and *μ* are not on the same sphere. We then define a kernel function on the domain *Ω* × *Ω* using a product of two functions *f*_*h*_ on *Ω*. That is, given a mean (*μ*_*x*_, *μ*_*y*_) the kernel is defined as *k*_*h*_((*x*,*y*); (*μ*_*x*_, *μ*_*y*_)) = *f*_*h*_(*x*, *μ*_*x*_)*f*_*h*_(*y*, *μ*_*y*_).

The bandwidth of the kernel function is a key parameter under KDE. Although many bandwidth selection criterion have been proposed (Botev, Grotowski, & Kroese, 2010; A. W. Bowman, 1984; C. Jones, Marron, & Sheather, 1996; M. C. Jones, Marron, & Sheather, 1996; Moyer et al., 2017; Risk & Zhu, 2019; Turlach, 1993, January; Zhengwu Zhang, Klassen, & Srivastava, 2019), there is no consensus on the best general approach. In this paper, we select *h* based on the reproducibility of ${\hat{C}}_{\mathrm{SC}}\left(x,y\right)$ calculated on the HCPTR dataset.

### 2.5 SC‐FC coupling

We are ready to study the integration of continuous SC and FC defined on the domain *Ω* × *Ω*. We define three novel definitions of SC‐FC coupling (SFC) based on our continuous SC and FC.

***ContinuousglobalSC‐FCcoupling***: We first evaluate the consistency between SC and FC on the surface without any predefined parcellation of the brain. Let $C_{\mathrm{SC}}\left(x,y\right)$ and $C_{\mathrm{FC}}\left(x,y\right)$ denote the continuous SC and FC for a particular subject. At any point *x*_0_ in *Ω*, we define the global SC‐FC coupling using a normalized inner product of two functions $f_{1}\left(y\right)=C_{\mathrm{SC}}\left(x_{0},y\right)$ and $f_{2}\left(y\right)=C_{\mathrm{FC}}\left(x_{0},y\right)$:

(4) $$\mathrm{SF}C_{\mathrm{gbl}}\left(x_{0}\right)=\langle \frac{f_{1}}{∥f_{1}∥},\frac{f_{2}}{∥f_{2}∥}\rangle =\frac{\int_{s\in \Omega }C_{\mathrm{SC}}\left(x_{0},s\right)C_{\mathrm{FC}}\left(x_{0},s\right)\mathrm{ds}}{\sqrt{\int_{s\in \Omega }C_{\mathrm{SC}}^{2}\left(x_{0},s\right)\mathrm{ds}\int_{s\in \Omega }C_{\mathrm{FC}}^{2}\left(x_{0},s\right)\mathrm{ds}}}$$

The inner product 〈,〉 is defined for two functions on *Ω* and is analogous to calculating the correlation between two rows of a functional connectivity and structural connectivity matrix in a traditional setting.

**FIGURE 3:**
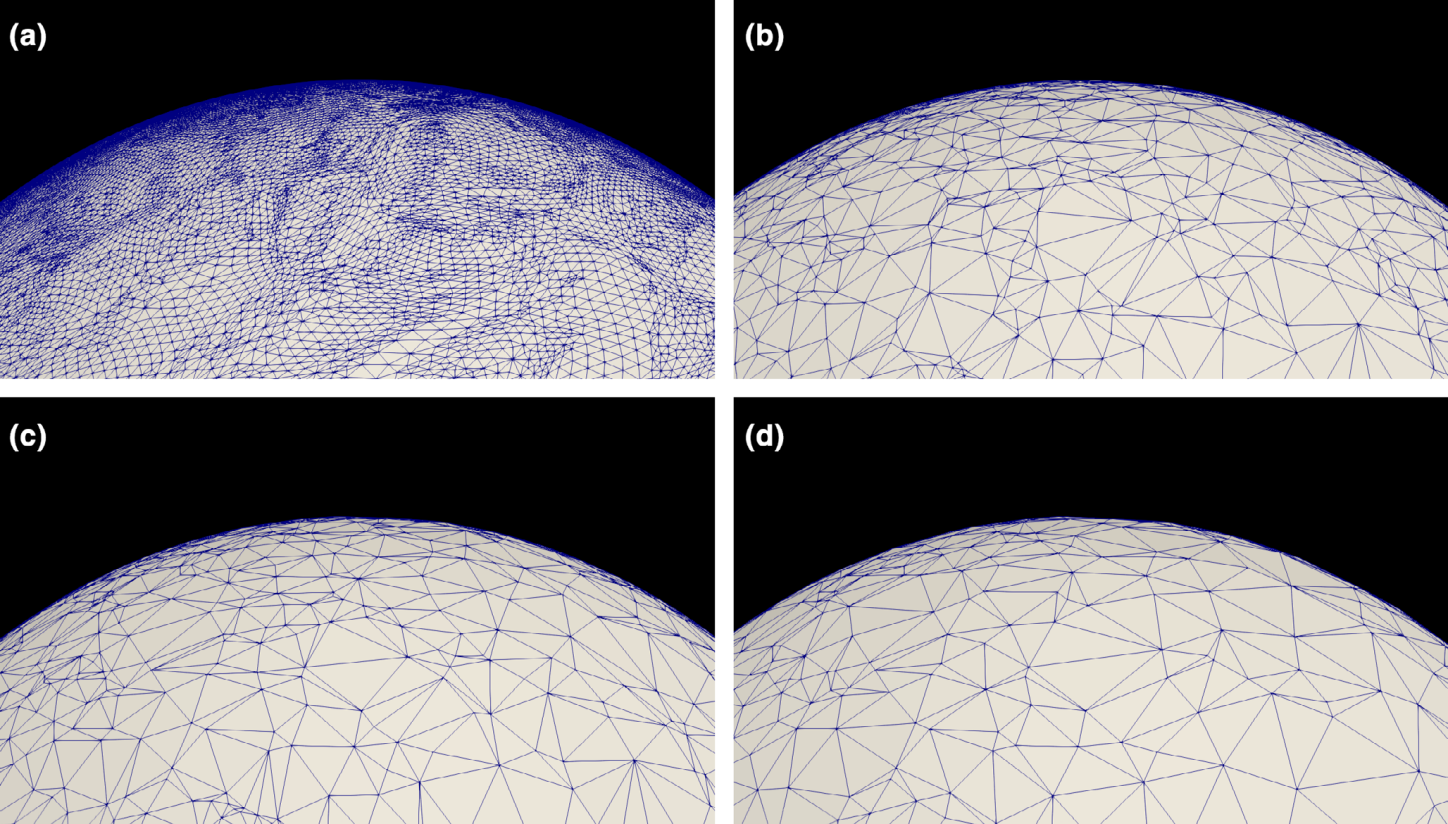
Example grids of *Ω* in SBCI. From (a) to (d), we have different sparsity levels of vertices on a 2‐sphere: (a) 163,842 vertices; (b) 8,453 vertices; (c) 5,157 vertices; and (d) 1,834 vertices. The down‐sampling is conducted by removing vertices that induce the least error (measured by the Hausdorff distance) between the resulting mesh and the full resolution mesh

The *SFC*_*gbl*_(*x*_0_) returns a scalar at *x*_0_, and therefore, *SFC*_*gbl*_ is a continuous function on *Ω*, measuring the similarity/consistency between SC and FC at different locations on the white surface. In practice, we evaluate the *SFC*_*gbl*_ on a discrete grid of *Ω*. The mesh surfaces from Freesurfer provide a natural choice for such a grid. However, the dense vertices on these surfaces cause computational challenges. In our implementation of SBCI, we down‐sample each white surface mesh (left and right) from over 120,000 vertices to around 2,100 using the Visualization Toolkit (VTK) in Python (Schroeder, Martin, & Lorensen, 2006; Schroeder, Zarge, & Lorensen, 1992). To maintain as much topological information as possible, we sample vertices from the white surface such that the induced Hausdorff distance (Aspert, Santa‐Cruz, & Ebrahimi, 2002) between the full and down‐sampled meshes is minimized. We then generate the down‐sampled white, inflated, and spherical surface meshes using Delaunay triangulation (Barber, Dobkin, & Huhdanpaa, 1996) and the coordinates corresponding to the sampled vertices on the full meshes. Figure 3 shows a surface mesh before (panel (a)) and after down‐sampling at three different sparsity levels of vertices (panels (b), (c) and (d)). With the down‐sampled mesh in (d), in the experiment section, our final continuous SC and FC are represented with matrices of 3668 × 3668 dimensions before masking out the corpus callosum region.

***ContinuouslocalSC‐FCcoupling***: When a parcellation of the brain surface is available, we can evaluate the coupling strength of our continuous SC and FC within ROIs. For an ROI *E* and a point *x*_0_ ∈ *E*, our continuous local SFC (*SFC*_*loc*_) is defined as:

(5) $$\mathrm{SF}C_{\mathrm{loc}}\left(x_{0}\right)=\langle \frac{f_{1}^{E}}{∥f_{1}^{E}∥},\frac{f_{2}^{E}}{∥f_{2}^{E}∥}\rangle =\frac{\int_{s\in E}C_{\mathrm{SC}}\left(x_{0},s\right)C_{\mathrm{FC}}\left(x_{0},s\right)\mathrm{ds}}{\sqrt{\int_{s\in E}C_{\mathrm{SC}}^{2}\left(x_{0},s\right)\mathrm{ds}\int_{s\in E}C_{\mathrm{FC}}^{2}\left(x_{0},s\right)\mathrm{ds}}}.$$

***Discreteparcellation‐basedSC***, ***FCandSC‐FCcoupling***: At last, following existing literature (Cocchi et al., 2014; Jiang et al., 2019; J. Wang et al., 2018; Zhiqiang Zhang et al., 2011), we define discrete SFC for a given parcellation. We first convert our continuous SC and FC to finite adjacency matrices based on the given parcellation. For any two ROIs *E*_1_ and *E*_2_, we define the SC between the two regions as:

(6) $$\mathrm{SC}\left(E_{1},E_{2}\right)=\frac{\iint_{x\in E_{1},y\in E_{2}}C_{\mathrm{SC}}\left(x,y\right)\text{dxdy}}{\left|E_{1}\right|\left|E_{2}\right|},$$

where ∣*E*_*i*_∣ represents the area of region *E*_*i*_ for *i* = 1, 2. The defined SC strength represents the connectivity density in a unit area square.

The traditional way to calculate FC is to first obtain a mean BOLD signal for each region and then calculate the Pearson correlation coefficient. To be more consistent with the SC calculation, we instead consider an average of the correlations. We calculate the FC in the following way: first apply the Fisher *z*‐transformation to the correlation, calculate the average, and then apply the inverse Fisher *z*‐transformation:

(7) $$\mathrm{FC}\left(E_{1},E_{2}\right)=\tanh\left(\frac{\iint_{x\in E_{1},y\in E_{2}}\text{artanh}\left(C_{\mathrm{FC}}\left(x,y\right)\right)\text{dxdy}}{\left|E_{1}\right|\left|E_{2}\right|}\right),$$

where tanh(·) and artanh(·) represent the hyperbolic tangent function and its inverse respectively. Finally, we define the discrete SC‐FC coupling (*SFC*_*dct*_) as:

(8) $$\mathrm{SF}C_{\mathrm{dct}}\left(E\right)=\text{corr}\left(\mathrm{SC}\left(E,\cdot \right),\mathrm{FC}\left(E,\cdot \right)\right)$$

where *corr*(·, ·) represents the Pearson correlation and *SC*(*E*, ·) and *FC*(*E*, ·) represent the structural and functional connections between ROI *E* and all other ROIs respectively.

### 2.6 Evaluation and analysis

To construct and validate the SBCI pipeline, we performed the following analyses. Note that in our experiments, a few parcellation approaches were involved: atlas‐free (our continuous SC, FC, and SFC), the Desikan–Killiany (Desikan et al., 2006) atlas (68 cortical ROIs), the Destrieux (Destrieux, Fischl, Dale, & Halgren, 2010) atlas (148 cortical ROIs), and the Brainnetome (Fan et al., 2016) atlas (210 cortical ROIs). We did not consider any connections to subcortical regions.

#### 2.6.1 SBCI parameter selection

A few parameters in SBCI are critical to connectome mapping: the surface flow size *t* in SET, the number of tractography seeds *N*_*s*_, and the SC smoothing bandwidth *h*. Using the HCPTR dataset, we optimized these parameters based on the reproducibility of our final SC. The reproducibility is measured using the distance‐based intraclass correlation coefficient (dICC), defined as $\text{dICC}={\bar{d}}_{\mathrm{bs}}^{2}/\left({\bar{d}}_{\mathrm{bs}}^{2}+{\bar{d}}_{\mathrm{ws}}^{2}\right)$ (Zhengwu Zhang et al., 2018), which is a generalization of the intraclass correlation coefficient (ICC) (Shrout & Fleiss, 1979), with values in the range (0, 1). ${\bar{d}}_{\mathrm{bs}}^{2}$ and ${\bar{d}}_{\mathrm{ws}}^{2}$ represent the average distance squared between subjects and within multiple scans of a subject respectively. The dICC is calculated under the Frobenius norm using the entire connectivity matrix. Higher dICC values indicate better reproducibility.

The surface smoothing kernel FWHM *σ* is another parameter in SBCI that can be tuned for the functional data. We selected *σ* = 5 mm to remain consistent with typical fMRI preprocessing procedures.

#### 2.6.2 SBCI connectome reproducibility

To validate the SBCI pipeline, we performed qualitative and quantitative exploratory analyses to assess the reliability of our pipeline and compared them to previous studies. These analyses fall into two categories: SC and FC reproducibility and the relationship between SC and FC. Comparisons were made using the atlas‐free approach and atlas‐based approaches. With the atlas‐free approach, we performed visual inspections of SC and FC at different spatial resolutions (different grids on *Ω*, refer to Figure 3) and calculated the ICC and dICC using the HCPTR dataset. While ICC produces a value at every vertex, allowing for the calculation of summary statistics of the distribution, dICC produces only a single value per connectome. As such, to account for variability we obtained 10,000 bootstrap samples (randomly sampling 36 subjects with replacement) and took the median dICC value and interquartile range (IQR). IQR is defined as the difference between the 75th and 25th percentiles. Given the bootstrap results, we also calculated the *p*‐value *P*(dICC < 0.5), where dICC < 0.5 indicates that the measurement is not reproducible. Note that in each test and retest scanning session in the HCPTR dataset, we had one dMRI scan and four resting state fMRI runs, resulting in a total of 72 SC matrices and 288 FC matrices for the entire dataset.

#### 2.6.3 SC‐FC coupling reproducibility

After confirming the validity of our SBCI pipeline to produce consistent and reproducible SC and FC at both standard atlas and atlas‐free resolutions, we sought to examine the reproducibility of the SFC features. After registering each subject's images to the standard fsaverage space, we obtained aligned SC and FC on a common white surface. Using the SFC definitions presented in Section 2.5, we quantified the reproducibility of each feature using both ICC and dICC measures. For each subject in the HCPTR dataset, we had 2 SC matrices and 4 FC matrices, and therefore obtained 8 (= 2  × 4) SFC measures for each definition of SFC for each subject, resulting in 288 (= 8  × 36) total features for all 36 subjects. The five SFC features considered in this paper were: global SC‐FC coupling (*SFC*_*gbl*_; Equation (4)); local SC‐FC coupling based on the Desikan–Killiany atlas (*SFC*_*locdk*_; Equation (5)); local SC‐FC coupling using major brain lobes (*SFC*_*loclb*_; Equation (5)); discrete SC‐FC coupling based on different atlases (*SFC*_*dct*._) defined by Equation (8); and traditional discrete SC‐FC coupling (*SFC*_*trd*.._), where SC is calculated based the summation of streamlines in a connection and FC is calculated based on the Pearson correlation of the mean BOLD signals in each ROI pair.

Note that for *SFC*_*loclb*_ the lobar parcellation was created by merging ROIs within the Desikan–Killiany atlas to achieve 12 larger areas (right and left frontal, parietal, temporal and occipital lobes, and the right and left insula and cingulate).

#### 2.6.4 SC‐FC coupling as imaging markers

In order to assess the usefulness of the SFC features as imaging markers, we used the HCPYA dataset, described in Section 2.1, to find group differences due to sex. We first performed independent *t*‐tests on the surface. A nonparametric suprathreshold cluster test (Nichols & Holmes, 2002) was conducted to determine significant regional differences between the two groups. Clusters were defined as those regions of neighboring vertices at which the uncorrected *p*‐values (from the *t*‐tests) between the two groups were ≤ 0.05. We then generated a permutation distribution for the maximal cluster size and determined a critical suprathreshold cluster size with threshold *p* ≤ 0.05. All vertices within a significantly large cluster were deemed to be significant. Suprathreshold cluster tests like this are generally more powerful for neuroimaging data than techniques such as Bonferroni adjustment and Benjamini–Hochberg false discovery rate control (Friston et al., 1994; Nichols & Holmes, 2002).

We also conducted a classification analysis to compare the discriminative abilities of different SFC features in distinguishing males from females. Principle component analysis (PCA) was first performed on the training data and applied to the test data to reduce the dimensionality of each SFC feature before different classifiers were applied. To make a robust comparison, results were obtained from different classifiers including the logistic regression classifier (LRC), support vector classifier (SVC), and random forest classifier (RFC). Repeated five fold cross‐validation was performed (100 times), tuning hyperparameters through nested cross‐validation, and the receiver operator characteristic (ROC) area under the curve (AUC) was used to evaluate the classification performance for each iteration.

## 3 RESULTS

### 3.1 Parameter selection

We selected *t* = 75 and *N*_*s*_ = 3 × 10^6^ for all subsequent experiments. Details are presented in Data S1. The flow parameter *t* = 75 maximizes the dICC and thus SC reproducibility regardless of the number of seeds used for every approach except the Desikan–Killiany atlas. The number of seeds selected was determined based on a diminishing return of dICC as a function of computational resources.

We then sought an optimal value for *h* to maximize the dICC of the atlas‐free approach. Table 1 shows dICC values for the four approaches with five different KDE bandwidth values, *h* ∈ {0, 0.002, 0.005, 0.01, 0.02} after removing two outliers. As our pipeline is designed to conduct connectivity analyses using an atlas‐free approach, we selected *h* = 0.005 because it maximized the dICC value when using the atlas‐free approach and was still close to maximizing the atlas‐based approaches. We found that smoothing was especially helpful for enhancing the reproducibility of high resolution SC. In fact, after smoothing, the dICC for our atlas‐free approach was nearly the same as all three parcellations, which is a substantial improvement over the original unsmoothed SC (*h* = 0). Using an Intel CPU (2.70 GHz), it took approximately 8, 4, and 2 hrs to smooth SC matrices obtained using grids *Ω* of 16,906, 10,314, and 3,668 vertices, respectively, producing SC matrices requiring 1.1GB, 0.5GB, and 0.1GB of storage. The full SBCI pipeline took approximately 4–5 days (10 hrs for fMRI processing, 1 day for T1w processing, 2–3 days for dMRI processing, and 12 hrs for connectome integration) to run for a single subject.

**TABLE 1:** Reproducibility measure dICC vs. bandwidth for the continuous SC

Atlas	*h* = 0	*h* = 0.002	*h* = 0.005	*h* = 0.010	*h* = 0.020
Desikan	0.80	**0.81**	**0.81**	0.80	0.80
Destrieux	0.79	0.78	0.79	**0.80**	**0.80**
Brainnetome	0.79	0.80	**0.81**	**0.81**	**0.81**
Atlas‐free	0.75	0.79	**0.80**	0.79	0.79

*Note*: The table shows results after removing outliers when using three atlases and the atlas‐free approach with different KDE bandwidth parameters (*h*). The parameters with the best dICC are in bold for each approach.

### 3.2 Exploratory connectome analyses

**FIGURE 4:**
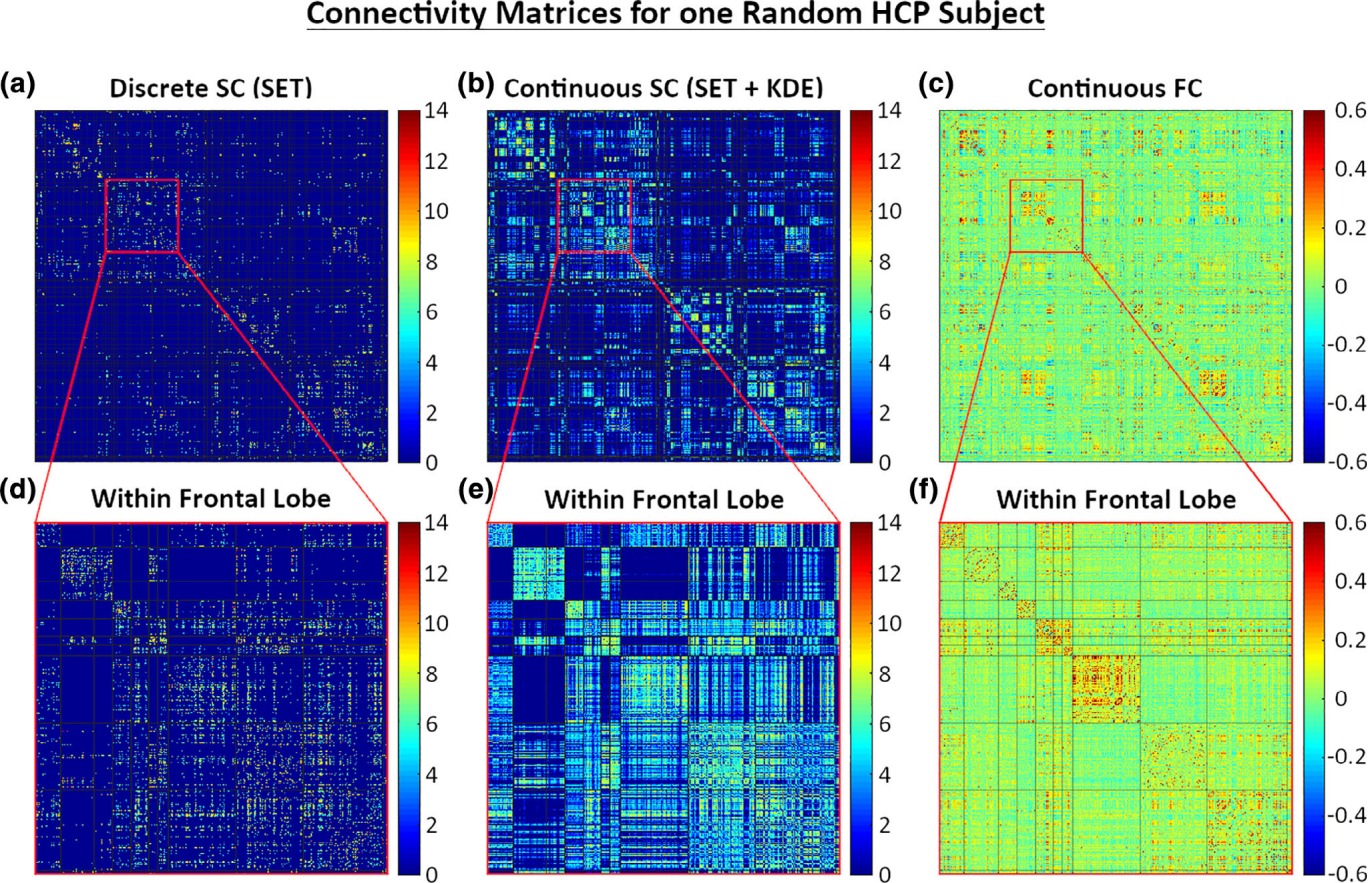
Outputs from SBCI. (a) Discrete SC before smoothing; (b) continuously smoothed SC using *h* = 0.005; (c) Continuous FC; and (d–f) zoomed in on the left frontal lobe for connectomes in (a–c). The black horizontal and vertical lines in (e–f) designate different ROIs in the Desikan–Killiany atlas for comparison with typical atlas‐based connectivity matrices. We multiplied a constant to the continuous SC in (b) and (e) for visualization purposes

Figure 4 shows SC and FC matrices for one randomly selected subject, comparing different types of SC and their sparsity levels to the continuous FC. The sparsity (defined as the percentage of 0 elements) of each connectome was 0.97 and 0.55 for the discrete and continuous SC, respectively. As we can see, the continuous SC is much more dense after smoothing.

**FIGURE 5:**
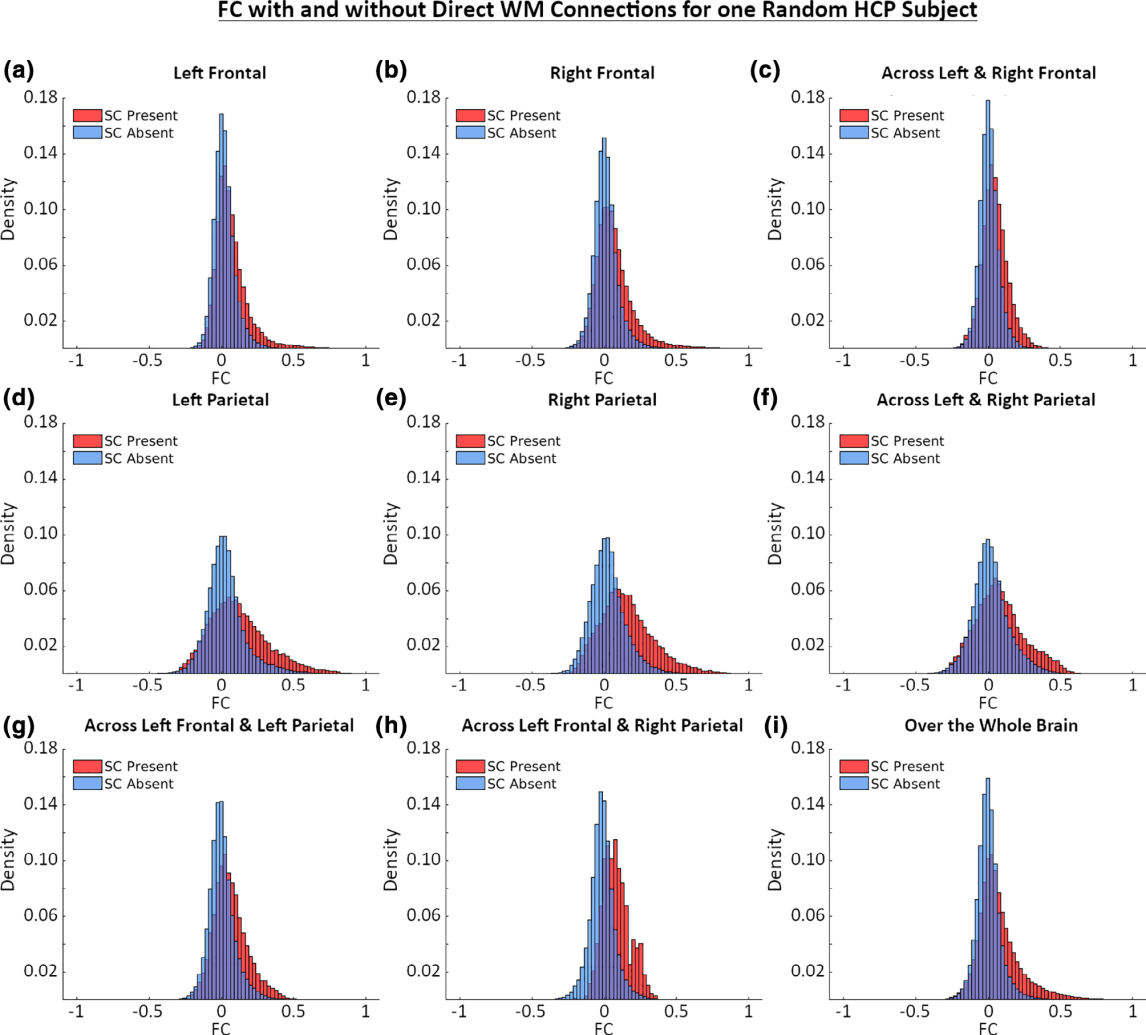
Histograms of resting state FC for nodes‐pairs with and without direct WM SC connections. Elements from the FC matrix were assigned to one of two groups depending on whether or not there is a value > 10^−7^ present at the corresponding element in the SC matrix. The histograms show the distribution of FC strength for both of these groups using only those connections within a region (a, b, d, and e) (defined by the Desikan–Killiany atlas) or only those connections shared across regions (c, f, g, and h). Finally, (i) shows the histogram for the whole brain using every connection over the entire surface

Next, we evaluated the relationship between SC and FC. In Figure 5, we show the histograms of FC strengths with and without direct SC connections. In our continuous SC framework, we defined a direct SC connection as those node‐pairs with SC values greater than 10^−7^. This means if ten million streamlines were built for a subject, the connections that had more than one expected streamline were considered to have a direct SC connection. We found that FC strengths between regions with direct SC is consistently higher than FC strengths between regions without direct SC connections (*p*‐value < 10^−6^).

Finally, we quantitatively assessed the reproducibility of SC and FC produced by SBCI. We compared the dICC and node‐wise ICCs between two parcellation approaches: Desikan–Killiany and atlas‐free. The median (IQR) dICC values of SCs derived from the Desikan–Killiany and atlas‐free approaches were 0.80 (0.03) and 0.79 (0.01) respectively. The median (IQR) dICC values of FC for Desikan–Killiany and atlas‐free approaches were 0.60 (0.01) and 0.61 (0.01) respectively. All dICC values were significant with *p*‐values less than 10^−6^. We also examined the reproducibility of individual connections by calculating node‐wise ICC values. Structural ICC distributions had medians (IQR) 0.74 (0.17) and 0.69 (0.27) for the Desikan–Killiany and atlas‐free approaches respectively. Functional ICC distributions had medians (IQR) 0.30 (0.20) and 0.27 (0.25) for the Desikan–Killiany and atlas‐free parcellations.

### 3.3 SC‐FC coupling reproducibility

We then evaluated the reproducibility of our SFC features. Figure 7 shows the pairwise distance matrices, dICC, and ICC histograms for the six different SFC features. The median (IQR) dICC values of the continuous SFC features *SFC*_*gbl*_, *SFC*_*locdk*_, and *SFC*_*loclb*_ were 0.68 (0.01), 0.72 (0.01), and 0.70 (0.01) respectively. All *p*‐values were less than 10^−6^. Most of the ICC values of the global and local SFC features fall in the fair to good reliability range. In contrast, the median (IQR) dICC values of the discrete SFC features *SFC*_*dctdk*_ (Desikan), *SFC*_*dctds*_ (Destrieux), and *SFC*_*dctbr*_ (Brainnetome) were 0.60 (0.01), 0.61 (0.01), and 0.62 (0.01) respectively. All *p*‐values were less than 10^−6^. The ICC values for the discrete SFC features were all in the poor reliability range.

**FIGURE 6:**
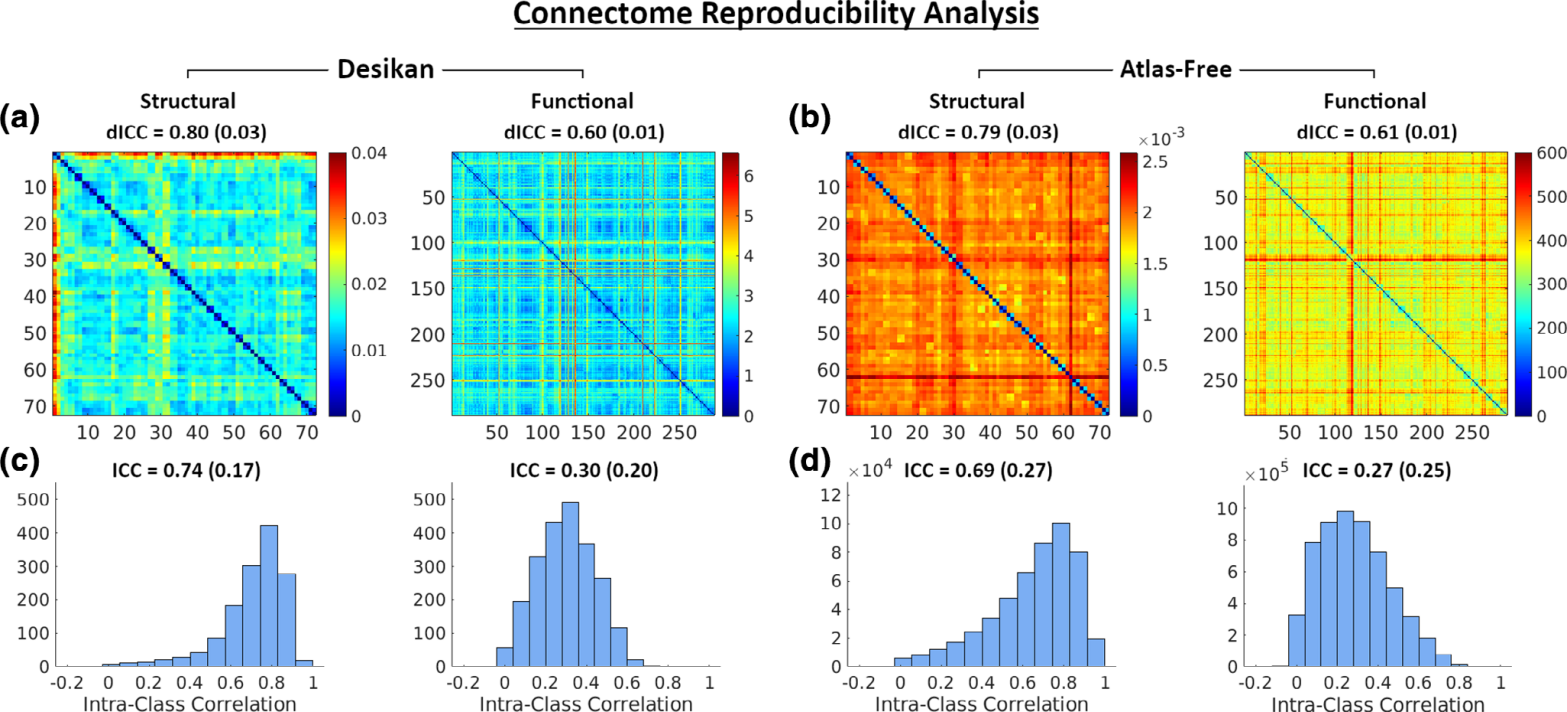
Reproducibility analysis of SC and FC from SBCI. The first row shows pairwise distance matrices of SCs and FCs generated under (a) the Desikan–Killiany atlas and (b) our atlas‐free approach. The Frobenius norm was used to calculate the distance between connectivity matrices, and the different scales are due to the differences in the connectivity matrices generated by each method (the relative difference between the intra‐subject distances and the intersubject distances is more important than the magnitude of those distances). Note that we had 72 SC and 288 FC matrices in the HCPTR dataset. Medians (IQR) from bootstrap sampling are displayed above each distance matrix. Panels (c) and (d) show ICC histograms for every node‐pair in the SCs and FCs corresponding to panels (a) and (b) respectively. Medians (IQR) are displayed above each histogram. We placed scans from the same subject next to each other, so that we could observe a block pattern along the diagonal of these distance matrices

**FIGURE 7:**
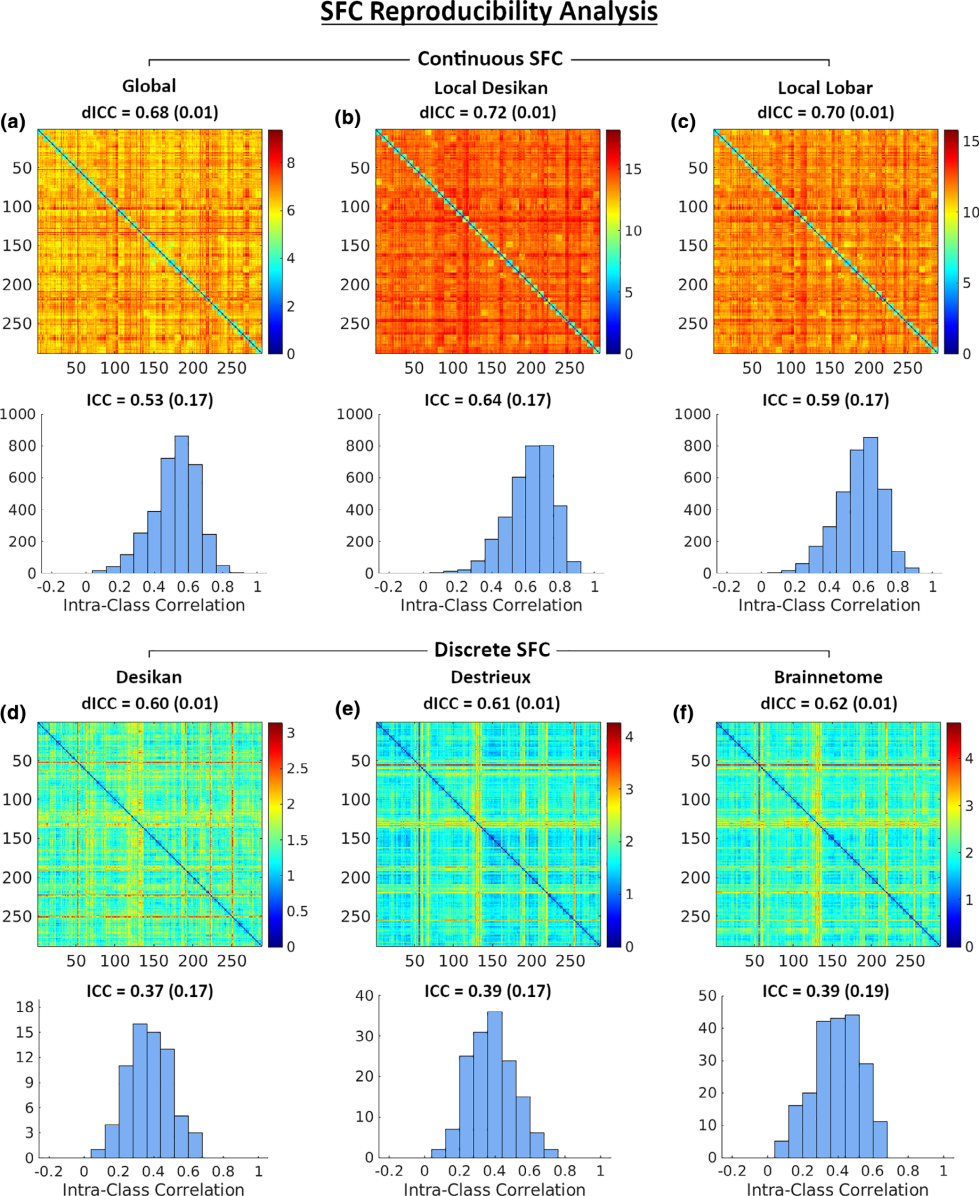
SFC reproducibility analysis. The first and third rows from left to right show pairwise distance matrices for the *SFC*_*gbl*_, *SFC*_*locdk*_, and *SFC*_*loclb*_ and the *SFC*_*dctdk*_ (Desikan), *SFC*_*dctds*_ (Destrieux), and *SFC*_*dctbr*_ (Brainnetome) features respectively. Medians (IQR) from bootstrap sampling are displayed above each distance matrix. The second and fourth rows show the corresponding histograms of ICC at each node. Medians (IQR) are displayed above each histogram

Comparing results in Figure 7 with the ones in Figure 6, we had a few interesting findings: (a) our continuous SFC features had much better reproducibility values (as measured by both ICC and dICC) compared to the FC; (b) the continuous local SFCs (representing the local SC‐FC association) were more reproducible than the global SFC (representing the global SC‐FC association); and (c) the discrete SFCs (*SFC*_*dct*_) were the least reproducible of all SFC measures. Plots of these SFC features for an individual can be found in Figures S3, S5, and S6. Our results show that the within‐subject *SFC*_*gbl*_, *SFC*_*locdk*_, *SFC*_*loclb*_, *SFC*_*dctdk*_, *SFC*_*dctds*_, and *SFC*_*dctbr*_ were reproducible (although to different degrees) in data from two sessions that occurred in the span of several months in healthy young adults, and the *SFC*_*gbl*_ was more variable between subjects than within subjects. Therefore, we expected the SFC features to be informative and robust markers to detect individual or group effects.

### 3.4 SC‐FC coupling sex difference

**FIGURE 8:**
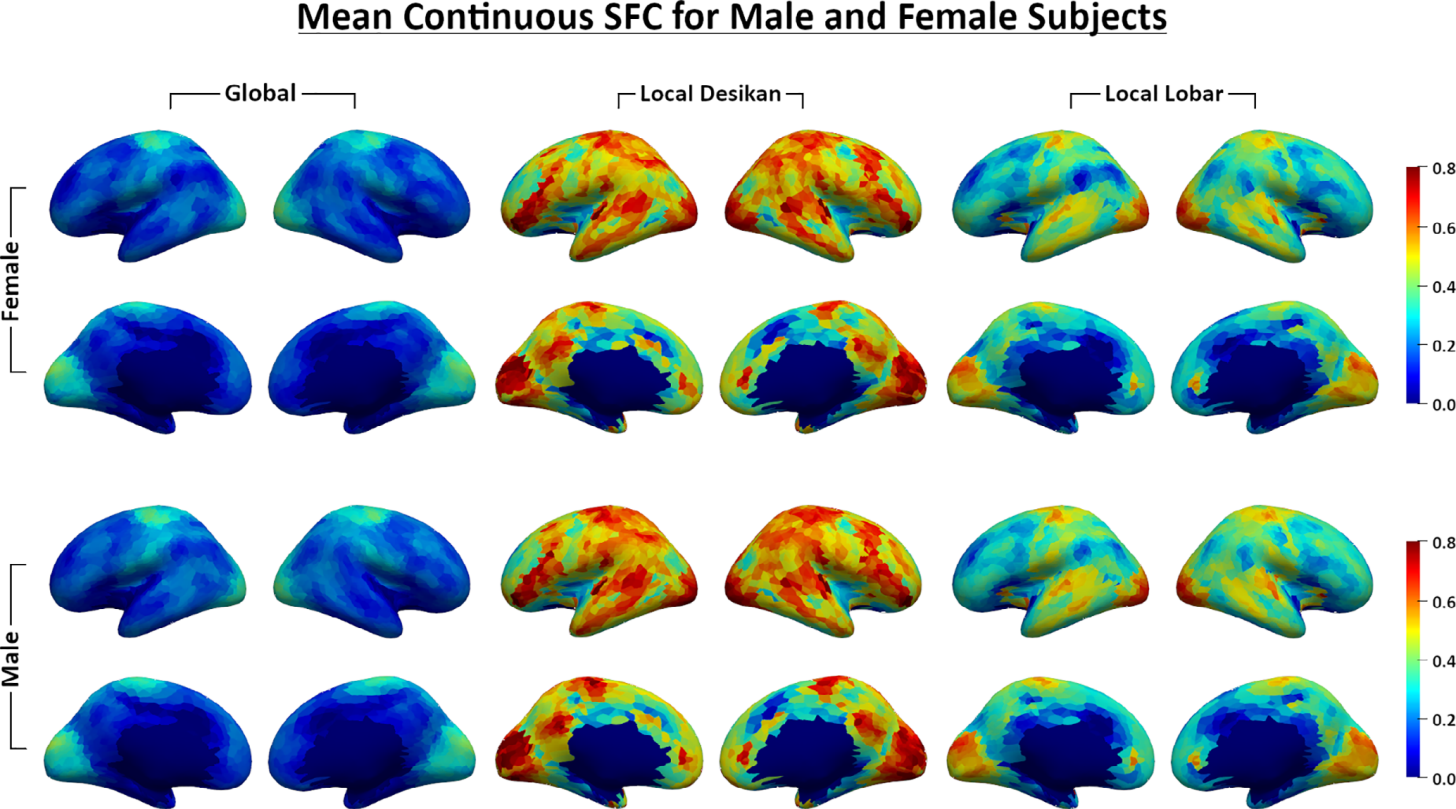
Mean *SFC*_*gbl*_, *SFC*_*locdk*_, and *SFC*_*loclb*_ values calculated over 43 male and 46 female subjects

**FIGURE 9:**
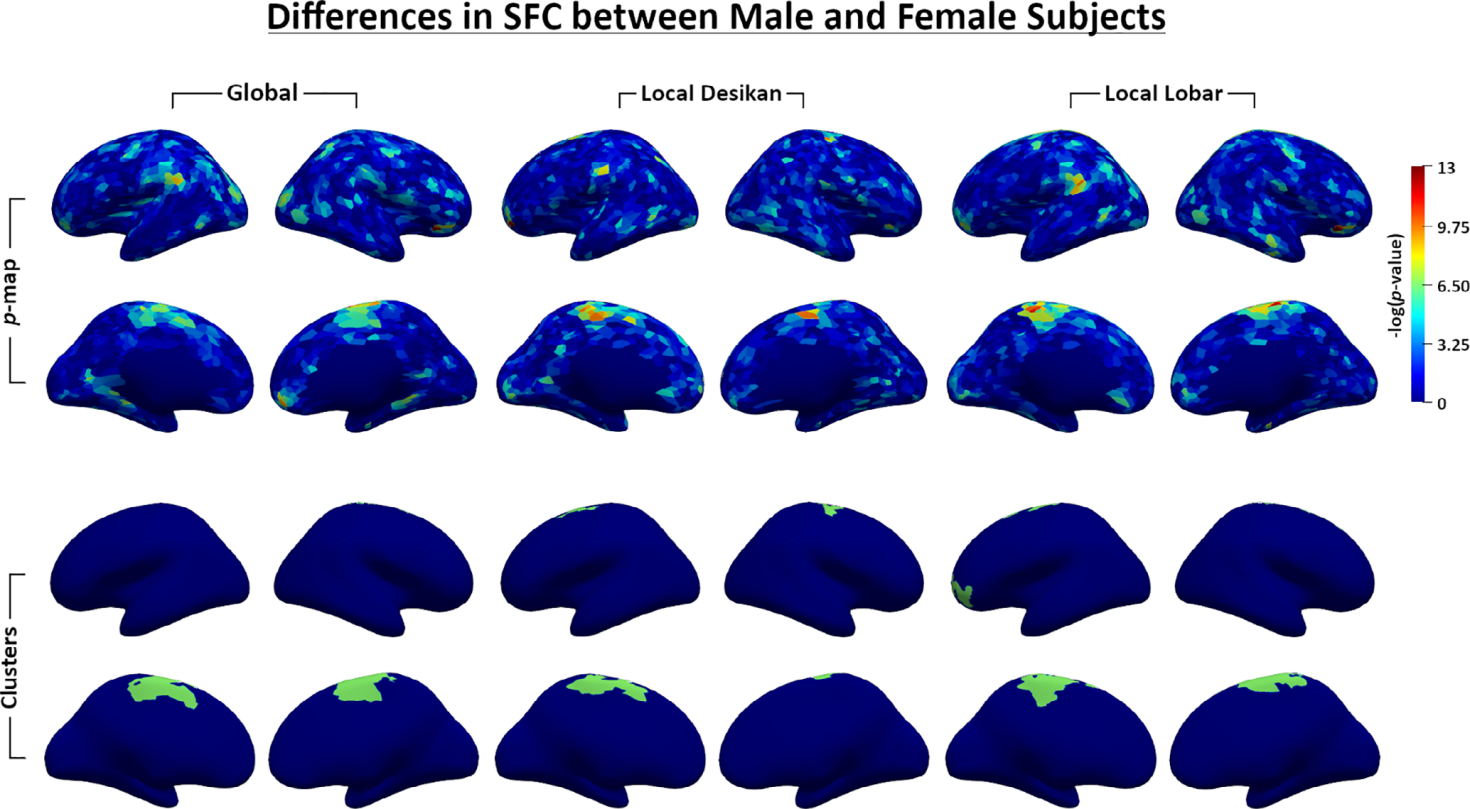
Results of testing differences in *SFC*_*gbl*_, *SFC*_*locdk*_, and *SFC*_*loclb*_ between two groups, one consisting of 43 male subjects, the other of 46 female subjects. The first two rows show the uncorrected negative log*p*‐values of *t*‐tests at every point on the surface for each of the SFC features. The second two rows show the significant suprathreshold clusters (thresholding with *p* ≤ 0.05)

Finally, we evaluated the usefulness of our SFC features as markers to detect group differences in sex. We used a small subset of the HCPYA data (due to high computational times and storage demand for each subject we have not processed all HCPYA subjects yet) containing 43 randomly selected males and 46 randomly selected females from the 26 to 30 year old group in the S500 data release. Figure 8 shows the average continuous (atlas‐free) SFC features (*SFC*_*gbl*_, *SFC*_*locdk*_, and *SFC*_*loclb*_) across all males and females on the inflated brain surfaces, and Figure S7 displays the average discrete SFC features (*SFC*_*dctdk*_, *SFC*_*dctds*_, and *SFC*_*dctbr*_). Additionally, using the three continuous SFC features, we conducted point‐wise independent *t*‐tests on the surface between males and females. Figure 9 shows the *p*‐values on the surface (the first two rows) and binary maps (the third and fourth rows) indicating significant differences after correcting for multiple comparisons based the nonparametric suprathreshold cluster test.

Table 2 shows the median and IQR of the AUCs from 100 repeated five fold stratified cross‐validation for each prediction model. Note that different classifiers and latent dimensions (denoted as *K* and obtained based on PCA) were used to provide a robust comparison across various choices of models and hyperparameter selections. We see that for nearly all classification models, the continuous SFC features were better at predicting sex than the discrete SFCs (both the novel and the traditional ones). The newly proposed discrete SFC features had much better performance compared with the traditional discrete SFC features. Table S3 shows the percent variance explained by different numbers of principle components for each SFC measure. For comparison, prediction results based on the continuous FC and SC connectomes are in Table S4.

**TABLE 2:** Sex prediction median_(*IQR*)_ AUC scores for different classification models with five fold stratified cross‐validation randomly repeated 100 times for various SFC measures

		Continuous SFC			Our discrete SFC			Traditional discrete SFC		
Model	*N*_*PCs*_	*SFC*_*gbl*_	*SFC*_*locdk*_	*SFC*_*loclb*_	*SFC*_*dctdk*_	*SFC*_*dctds*_	*SFC*_*dctbr*_	*SFC*_*trddk*_	*SFC*_*trdds*_	*SFC*_*trdbr*_
LRC	*K* = 10	0.81_(0.04)_	0.86_(0.03)_	**0.89**_(0.03)_	0.77_(0.03)_	0.71_(0.04)_	0.69_(0.04)_	0.62_(0.04)_	0.66_(0.04)_	0.68_(0.04)_
	*K* = 15	0.82_(0.03)_	0.84_(0.03)_	**0.86**_(0.04)_	0.74_(0.04)_	0.69_(0.04)_	0.76_(0.04)_	0.63_(0.04)_	0.69_(0.04)_	0.70_(0.04)_
	*K* = 20	**0.84**_(0.04)_	0.81_(0.03)_	0.83_(0.04)_	0.73_(0.04)_	0.73_(0.04)_	0.83_(0.03)_	0.66_(0.05)_	0.66_(0.04)_	0.70_(0.05)_
SVC	*K* = 10	0.81_(0.03)_	**0.84**_(0.03)_	**0.84**_(0.03)_	0.77_(0.03)_	0.71_(0.04)_	0.66_(0.05)_	0.48_(0.08)_	0.62_(0.08)_	0.66_(0.05)_
	*K* = 15	**0.86**_(0.03)_	0.82_(0.03)_	0.85_(0.05)_	0.73_(0.04)_	0.71_(0.04)_	0.78_(0.05)_	0.58_(0.09)_	0.63_(0.06)_	0.67_(0.04)_
	*K* = 20	**0.81**_(0.04)_	0.78_(0.05)_	**0.81**_(0.04)_	0.73_(0.05)_	0.70_(0.04)_	0.80_(0.03)_	0.62_(0.07)_	0.62_(0.05)_	0.67_(0.05)_
RFC	*K* = 10	0.72_(0.03)_	**0.80**_(0.03)_	0.77_(0.04)_	0.69_(0.04)_	0.66_(0.05)_	0.76_(0.04)_	0.63_(0.05)_	0.63_(0.04)_	0.71_(0.05)_
	*K* = 15	0.79_(0.04)_	**0.81**_(0.03)_	0.77_(0.05)_	0.73_(0.05)_	0.67_(0.03)_	**0.81**_(0.04)_	0.64_(0.04)_	0.64_(0.04)_	0.74_(0.04)_
	*K* = 20	0.74_(0.05)_	0.82_(0.04)_	0.76_(0.04)_	0.76_(0.04)_	0.68_(0.05)_	**0.84**_(0.04)_	0.59_(0.05)_	0.67_(0.04)_	0.73_(0.04)_

*Note*: K corresponds to the number of principal component scores used for dimension reduction. The SFC features with the best median predictive power are in bold for each classification model. Subscripts denote the type of SFC {gbl, global (Equation (4)); loc, local (Equation (5)); dct, discrete (Equation (8)); trd, traditional (defined as the correlation of the streamline count (SC) and correlation of the mean BOLD signals (FC) between two regions)} and the corresponding parcellated atlas {dk, Desikan–Killiany; lb, Lobar; ds, Destrieux; br, Brainnetome}.

Abbreviations: LRC, logistic regression classifier; SVC, support vector classifier; RFC, random forest classifier.

## 4 DISCUSSION

In this paper, we developed a novel atlas‐free approach for studying connectivity integration. The proposed SBCI framework can extract reproducible and discriminative high‐resolution structural connectivity (SC) and functional connectivity (FC) from high‐quality MRI data on the white surface of the brain. Additionally, SBCI produces three novel subject‐level imaging markers that are reflective of the relationships between structural and functional brain signals. By using the HCP Test–Retest data, we showed that SBCI can build reproducible continuous SC, FC, and SFC measures. Further, using data from the HCP Young Adult study, we demonstrated that these novel continuous SFCs show greater discriminative power as markers, producing better clustering results than the typical atlas‐based SFC features.

### 4.1 Advantages of SBCI

SBCI uses a novel tractography algorithm (St‐Onge et al., 2018) together with the KDE smoothing technique (Moyer et al., 2017; Moyer, Gutman, Faskowitz, Jahanshad, & Thompson, 2016) to project both SC and FC to the white surface and extend the traditional definitions of SC and FC to a continuous framework. By treating SC and FC in a continuous fashion, we obtain high resolution SC and FC and avoid the need to rely on discrete brain parcellations to study connectivity. Further, by extending the typically sparse SC to be a continuous feature, we close the sparsity gap between SC and FC, allowing us to interrogate structural‐functional relationships more robustly, as FC is naturally more dense. This continuous treatment of SC also allows us to overcome some of the computational challenges that come with big data; we do not need to recover hundreds of millions of streamlines in order to obtain reproducible SC at high resolutions (refer to Table 1 and Table S3).

Another advantage of SBCI is that we can inspect SC‐FC relationships in local brain regions as well as across the entire cortical surface (Honey et al., 2009). Figure 5 shows that in general, FC connection strengths are higher between areas of the brain when direct SC connections are present compared to those without direct SC connections. The mean differences between the two types of FC are different for different brain regions, for example, the left and right parietal lobes (plots d and e) had larger differences than the left and right frontal lobes (plots a and b). More interestingly, we found that FC strengths without direct SC connections have a mean strength close to zero, while FC strengths with direct SC connections have a mean strength greater than zero. These patterns are consistently observed in all subjects in our study. As previously mentioned, published findings regarding how FC strength varies with SC is heterogeneous (Chamberland et al., 2017; Honey et al., 2009), implying that more care should be taken in the future to more completely examine such relationships.

Although the idea of dense FC and SC has been proposed before (e.g. the HCP promotes the use of brain mesh surfaces to analyze fMRI data (Coalson et al., 2018; Glasser, Coalson, et al., 2016) and Moyer et al. (2017) proposed a point process model to estimate continuous SC), continuous structural and functional connectome coupling has not been studied before to the best of our knowledge. In existing literature, using predefined parcellations to calculate discrete SFC is a standard choice (Baum et al., 2020; Buckner et al., 2013; Chamberland et al., 2017; Cocchi et al., 2014; Ghumman et al., 2016; Honey et al., 2009; Honey et al., 2010; Jiang et al., 2019) with almost no exceptions. From our results in Figures 7 and S7, and Table 2, we found that the discrete SFC measures have several drawbacks compared to our continuous SFC features: (a) they have low resolution (decided by the number of ROIs in the atlas); (b) they are less reproducible; and (c) they are less powerful to distinguish between groups. Our results demonstrate that SC and FC are more robustly integrated at higher resolutions. Finally, by introducing the continuous SFC, we introduce a large set of analysis tools for functional data analysis in mathematics and statistics to the field of brain network analysis. For example, we can define basis functions to represent the continuous SFC to significantly reduce dimensionality for subsequent statistical analysis and inference.

### 4.2 Continuous Connectome reproducibility

We found that the reproducibilities of our continuous connectomes are consistent at high resolution with previous atlas‐based studies. In general, we observed mostly good (0.75 ≥ ICC > 0.6) to excellent (ICC > 0.75) SC reproducibility for both the Desikan–Killiany parcellation and our atlas‐free approach. Our high resolution atlas‐free connectome reproducibilities as measured by the dICC were nearly on par with the Desikan–Killiany atlas‐based connectomes for both SC and FC, indicating that SBCI can produce high‐quality high resolution connectomes. Further, the maintenance of dICC values across both parcellation approaches for SC and FC data demonstrates that the within‐subject variations of each connectome were less than between‐subject variations regardless of spatial resolution. In comparison to SC, FC has poor (0.4 ≥ ICC) to fair (0.6 ≥ ICC > 0.4) reproducibility, with a majority of connections falling in the relatively poor reproducibility range. However, our FC reproducibility results are consistent with those presented in the literature using the same dataset (Tomasi, Shokri‐Kojori, & Volkow, 2017) and other processing pipelines (Noble et al., 2017). Compared with previous reproducibility studies (Noble, Scheinost, & Constable, 2019), SBCI maintains a typical FC reliability range even at very high spatial resolution.

### 4.3 Novel SC‐FC coupling features

In this work we proposed three novel continuous SFC features: *SFC*_*gbl*_, *SFC*_*loc*_, and *SFC*_*dct*_, defined by Equation (4), (5), and (8) respectively. The *SFC*_*gbl*_ characterizes how the SC between a given location and all other locations in the cortex relates to the FC at that location and all other locations. In discrete space, this is analogous to the correlation between a row of the FC matrix and the corresponding row of the SC matrix. In other words, both short and long range connections to the vertex will contribute to the final value of *SFC*_*gbl*_. The *SFC*_*loc*_ measures the similarity of SC and FC within a predefined region, bounded as defined by a given atlas. From the results in Figure 8, we saw that at most locations, *SFC*_*loc*_ is larger than *SFC*_*gbl*_, indicating that local SC and FC have much greater similarity within ROIs than global SC and FC. Finally, we defined the discrete SFC using the continuous SC and FC directly, deviating from the definitions most commonly presented in the literature, i.e., where SC is calculated by the summation of streamline counts and FC is calculated by the Pearson correlation of the mean BOLD signals between two regions.

From the first two columns in Figure 8, we found that the global SC and FC are more correlated in primary sensory/motor areas such as S1/M1 and the visual and auditory cortices and less correlated in secondary association areas like the prefrontal cortex. The spatial distribution of *SFC*_*gbl*_ seems to be consistent with the fundamental organizing architecture of the brain known since Brodmann's map was published in 1909 (Brodmann, 2007). Brodmann's work began with the idea that “specific physiological functions in the cerebral cortex depend on specific histological structure and connectivity.” This principle is clear in primary sensory areas where specific histological patterns and cortical layer structures are closely associated with functional activity (Zeki, 2016). More modern mapping identified the hierarchical map organization according to unimodal and multimodal association areas (Mesulam, 2000). Our global SFC feature peaks in sensory areas and gradually drop into the unimodal and multimodal association areas. These observations highlight the ability of our approach to advance brain mapping using modern data measurement techniques based on MRI.

### 4.4 SFC differences between males and females

The difference between the brains of males and females remains an interesting question that is largely unanswered. Past findings show that male and female brains have anatomical, functional, and biochemical differences (Weis et al., 2020; Zaidi, 2010). With SBCI, sex differences were observed most strongly in the ventro‐medial prefrontal cortex, the somatosensory‐motor areas, the supra‐marginal gyrus, and the occipitoparietal (before FDR) areas extending into the fusiform gyrus. These results emphasize how structure and function are differentially related between the sexes and are consistent with reported behavioral differences between men and women. For example, men and women perform differently on emotional recognition (Lausen & Schacht, 2018) and emotional decision making (van den Bos, Homberg, & de Visser, 2013), which is well known to engage the ventro‐medial prefrontal cortex (Bechara, Tranel, & Damasio, 2000). Men and women are also known to perform differently on facial recognition (Herlitz & Lovén, 2013), visual motion processing (S. O. Murray et al., 2018), and episodic memory recollection (Yonker, Eriksson, Nilsson, & Herlitz, 2003). Direct correlation between our measure of sex‐related SFC differences and cognitive‐behavioral differences between the sexes remains to be tested.

Our classification results (Table 2) demonstrate that the continuous SFC features were better at predicting sex than discrete SFCs regardless of base classification algorithm and number of PCs. Furthermore, the discrete SFC features based on the discretization of our continuous SFCs outperformed those calculated using the traditional definition of SFC. However, note that we only used the Desikan–Killiany atlas to calculate *SFC*_*loc*_ and three common atlases to calculate the discrete SFC features, which may not be optimal choices (Messé, 2019). Given the predictive powers of our SFC features to distinguish sex, we expect that these measures will also act as robust connectivity biomarkers for pathological applications, to be explored in future studies.

### 4.5 Future work

One limitation of the SBCI pipeline is that SET is sensitive to the input surfaces according to our results from the HCPTR dataset. SET uses the geometry of the surface a priori to initiate WM streamlines. If surface reconstructions are significantly different across two scanning sessions for a single subject, then SBCI is unlikely to produce reliable SC for that subject. As such, we removed two outliers as determined by the differences in white surface reconstructions between the test and retest scans for our reproducibility analyses. Future work for improving SBCI should also focus on faster and more robust surface reconstruction (e.g. better segmentation and surface reconstruction methods (Henschel et al., 2020; Zhao et al., 2019) or collecting and incorporating high resolution T2‐weighted (T2w) or fluid attenuated inversion recovery (FLAIR) images to the T1w processing pipeline (Glasser et al., 2013; Renvall, Witzel, Wald, & Polimeni, 2016; Van Essen et al., 2013; Zaretskaya, Fischl, Reuter, Renvall, & Polimeni, 2018). Further, the fMRI signal resampling and smoothing of SC were conducted on the spheres and the actual mapping between the white surfaces and spheres may introduce distortions. Another future work is to develop a more direct method where the resampling and SC smoothing are performed on the original white surfaces and compare it with the current spherical framework.

The computational resources required to perform SBCI, while not prohibitive, provide a challenge for analyzing large datasets. Future work will involve decreasing the total processing time. For example, deep learning has made it possible to reconstruct reliable surfaces in a fraction of the time required to perform recon_all (Henschel et al., 2020). Additionally, improvements to the KDE smoothing algorithm employed in this pipeline should also reduce processing time. As other faster diffusion and functional preprocessing pipelines (provided by FSL and Freesufer) become available, the computational cost accompanied by SBCI should decrease.

Finally, the use of subcortical structures is crucial to understanding the effects of various pathologies on SC and FC. As such, future developments to SBCI include extending both SC and FC to include subcortical regions in order to study functional networks beyond the cortical areas.

We have demonstrated that the SBCI pipeline can reliably reconstruct high resolution SC and FC and produce novel SFC features, removing the need of using predefined brain parcellations for connectomics studies that can potentially bias their results. Future work should use SBCI to explore pathological differences in various neurological diseases including HIV‐associated cerebral small vessel disease (CSVD), mild cognitive impairment (MCI), Alzheimer's disease (AD), and chronic pain, among others. Finally, we intend to use SBCI to determine an optimal brain parcellation for studying structural, functional, and structural‐functional coupling for individual and group effects.

## Supporting information


**Data S1** Supporting Information.

## Data Availability

The SBCI pipeline is publicly available at https://github.com/murrayk085/SBCI. Data can be downloaded from the Human Connectome Project with proper credentials. Details regarding exact data usage, including subject IDs used for each experiment, are described in the Supplementary Material.
